# Prognostic biomarker discovery via a connected network-constrained Cox proportional hazards model

**DOI:** 10.1093/bib/bbag055

**Published:** 2026-03-10

**Authors:** Lingyu Li, Wai-Ki Ching, Zhi-Ping Liu

**Affiliations:** Department of Biomedical Engineering, Shandong University, Jinan 250061, China; Department of Mathematics, The University of Hong Kong, Hong Kong, China; Department of Mathematics, The University of Hong Kong, Hong Kong, China; Department of Biomedical Engineering, Shandong University, Jinan 250061, China

## Abstract

Biomarker discovery in biomedical sciences can be framed as feature selection in machine learning [1]. However, existing methods often overlook gene co-localization within regulatory interaction networks, leading to the identification of isolated biomarkers with limited biological interpretability [2]. Here, we present the Connected Network-regularized Cox proportional hazards model (CNet-Cox), which incorporates network connectivity constraints into sparse regularization to identify prognostic biomarkers for breast cancer (BRCA) on the discovery dataset from TCGA (1,092 patients), while explicitly accounting for patient survival time. CNet-Cox reveals the network structures of prognostic genes, evaluated in the internal validation dataset with a concordance index of 0.913, surpassing traditional regularized Cox methods. CNet-Cox shifts biomarker recognition from isolated to connected features within biomolecular networks and offers new biological insights. Furthermore, we established a six-gene BRCA prognostic risk scoring (PRS) metric and validated its robustness across six independent external validation datasets comprising 1,829 patients, and one spatial transcriptomic dataset containing 4,992 spots. The PRS score consistently demonstrated superior performance in patient/sample stratification across extensive and diverse validation datasets. Overall, our comprehensive downstream analyses underscore that CNet-Cox offers a novel approach for embedding network topology into feature selection, enabling the systematic discovery of key connected prognostic biomarkers. This significantly advances early detection and prognosis prediction, facilitating precision medicine for BRCA.

**References**

1. Li L, Liu Z P. “Biomarker discovery from high-throughput data by connected network-constrained support vector machine.” Expert Systems with Applications 2023; 226: 120179.

2. Hartman E, Scott A M, Karlsson C, et al. “Interpreting biologically informed neural networks for enhanced proteomic biomarker discovery and pathway analysis.” Nature Communications 2023; 14(1): 5359.

